# Ho_2_O_3_-TiO_2_ Nanobelts Electrode for Highly Selective and Sensitive Detection of Cancer miRNAs

**DOI:** 10.3390/bios12100800

**Published:** 2022-09-27

**Authors:** Jingjie Cui, Xuping Wang, Shaowei Chen

**Affiliations:** 1 School of Automation, Hangzhou Dianzi University, Hangzhou 310018, China; 2 Department of Chemistry and Biochemistry, University of California, 1156 High Street, Santa Cruz, CA 95064, USA

**Keywords:** Ho_2_O_3_-TiO_2_, miRNA, detection, selective, sensitive, electrochemical

## Abstract

The design and engineering of effective electrode materials is critical in the development of electrochemical sensors. In the present study, Ho_2_O_3_-TiO_2_ nanobelts were synthesized by an alkaline hydrothermal process. The structure and morphology were investigated by X-ray diffraction (XRD) and field emission scanning electron microscope (FESEM) measurements. The Ho_2_O_3_-TiO_2_ nanobelts showed a distinctly enhanced (004) reflection peak and rough surfaces and were used for the electrochemical selective sensing of various cancer miRNAs, such as prostate cancer miR-141, osteosarcoma miR-21, and pancreatic cancer miR-1290. Voltammetric measurements showed an oxidation peak at +0.4, +0.2, and +1.53 V for the three different cancer biomarkers, respectively, with the detection limit as low as 4.26 aM. The results suggest that the Ho_2_O_3_-TiO_2_ nanobelts can be used as active materials to detect early cancers, for in vitro screening of anticancer drugs, and molecular biology research.

## 1. Introduction

Cancer is the most threatening disease in human life and health, and cancer research has been attracting extensive attention. As miRNAs are closely related to disease and it is generally believed that changes in nucleic acid molecules are the most critical primordial links of cell carcinogenesis, the research on miRNAs is important for the early diagnosis and immunotherapy of cancer [1]. For instance, recent studies have shown that miR-141 is a potentially useful biomarker for prostate cancer for the up-regulation in prostate cancer specimens [2]. MiR-21 plays an important role in developing osteosarcoma and can be used as a potential serum marker for it [3]. The content of miR-1290 will increase during the development of pancreatic cancer, so the determination of miR-1290 can be exploited for the early detection of pancreatic cancer [4]. However, current miRNA research and detection generally require complex pretreatment steps, expensive optical instruments, and fluorescent reagents [1]. Therefore, it is of great significance to develop rapid, sensitive, simple, and accurate miRNA detection methods that can be used for practical applications in life science research and medical and clinical diagnosis [5].

Nanomaterials have broad applications in the life sciences due to their unique physical and chemical properties and structural characteristics. Of these, titanium dioxide (TiO_2_) nanostructures have been exploited for the sensitive, accurate, rapid, and simple detection and diagnosis of cancer, e.g., nano-p–n junction heterostructures, NiO/TiO_2_ nanobelts for the detection of anticancer drugs and biointeractions with cancer cells [6], Au/TiO_2_ bi-heterostructured nanobelts for the specific sensing of diverse tumour cells [7], mesoporous TiO_2_ for the sensing of pancreatic cancer miR-1290 [8], rutile TiO_2_ facet heterojunction nanostructure nuclease for the detection of miR-155 [9], and TiO_2_ nanotube arrays for the detection of prostate protein antigen [10], among others.

For electrochemical biosensors, the active sensing material on the electrode acts as a catalyst that promotes the reaction of biochemical molecules to produce output signals [11]. The catalytic activity is the key factor in the selection and development of sensing materials which can be further manipulated by the doping of select rare earth elements [12] or the introduction of oxygen vacancies [13]. Holmium trioxide, Ho_2_O_3_, is an important rare earth metal (lanthanide) oxide with attractive electrical and optical properties. It has been widely used in environmental protection [14,15,16], functional ceramics [17,18], and sensing devices [19]. Mortazavi-Derazkola and coworkers [16] synthesized nano-Ho_2_O_3_ and observed an outstanding photocatalytic performance. Pan reported a high-k Ho_2_O_3_ sensing film for a pH ion-sensitive field-effect transistor (ISFET) [19]. Additionally, when Ho_2_O_3_ is doped into TiO_2_, Ti^4+^ is partly replaced by Ho^3+^, leading to the formation of abundant oxygen vacancies in TiO_2_ and hence enhancing bulk conductivity and surface electrocatalytic activity. This will ultimately improve the electrochemical sensing activity of TiO_2_. However, to the best of our knowledge, there have been no reports on using Ho_2_O_3_-TiO_2_ as active materials to detect cancer miRNAs.

In this work, Ho_2_O_3_-TiO_2_ nanobelts are prepared by using an alkaline hydrothermal process and exhibited high specificity and sensitivity for the electrochemical detection of cancer miRNAs. Specifically, Ho_2_O_3_-TiO_2_ nanobelts can selectively detect miR-141, miR-21, and miR-1290 at distinctly different potentials and achieve a limit of detection as low as 4.26 aM, which suggests that Ho_2_O_3_-TiO_2_ nanobelts may be used as a promising electrode material for early cancer diagnosis, drug screening, and molecular biology research.

## 2. Materials and Methods

### 2.1. Materials

Titania P-25 (TiO_2_, ca. 75 wt.% anatase and 25 wt.% rutile, Degussa Co., Frankfurt, Germany), Ho_2_O_3_, and sodium hydroxide, were purchased from China National Reagents Corporation Ltd. (Shanghai, China). MicroRNA oligonucleotides (cDNA) were purchased from TSINGKE, and purified by polyacrylamide gel electrophoresis (PAGE). Various target miRNAs (e.g., miR-1290, miR-141, miR-21) and related match or mismatch probes were mixed and heated at 90 °C for 2 min. The volume ratio of target miRNA to probe was 1:1. Samples were diluted to a final concentration of 0.9 µM hybridization solutions. RNase-free distilled water was used throughout all of the experiments.

### 2.2. Preparation of Ho_2_O_3_-TiO_2_ Nanobelts

Titanate nanobelts were synthesized via a hydrothermal process in a concentrated NaOH aqueous solution. Briefly, 0.1 g of the P-25 precursor and 9 mg of the Ho_2_O_3_ powders were mixed with 20 mL of a 10 M NaOH aqueous solution, and underwent a hydrothermal treatment at 180 °C in a 25 mL Teflon-lined autoclave for 72 h. The resulting powders were then immersed in a 0.1 M HCl aqueous solution for 24 h and washed thoroughly with de-ionized water. Finally, the collected samples were dried and calcined at 600 °C for 2 h to obtain Ho_2_O_3_-TiO_2_ nanobelts.

### 2.3. Structure Characterization

A Bruker D8 Advance powder X-ray diffractometer was used to investigate Ho_2_O_3_-TiO_2_ nanobelts (a Cu-Kα source, λ = 0.15406 nm) at room temperature within the range of 2θ = 10 to 70°. Field-emission scanning electron microscopy (Hitachi S-4800) was used to characterize the morphology and size of the synthesized Ho_2_O_3_-TiO_2_ nanobelts. 

### 2.4. Electrochemistry Tests

The working electrode is assembled as follows: a glassy carbon electrode (GCE, 3 mm diameter) was polished with 0.05 µm Al_2_O_3_ suspensions and rinsed extensively with anhydrous ethanol and de-ionized water, and then electrochemically cleaned in 0.5 M H_2_SO_4_ by cycling potentials between −0.3 and +1.8 V at 100 mV·s^−1^. A nafion adhesive and an ethanol suspension of Ho_2_O_3_-TiO_2_ nanobelts (0.5 mg mL^−1^) were drop-cast onto the cleaned GCE surface. After drying, the resulting electrodes were used as sensing electrodes.

Electrochemical measurements were performed in a three-electrode configuration. The working electrode is the Ho_2_O_3_-TiO_2_ nanobelts-modified electrodes prepared above, a counter electrode (Pt foil with a geometric area of 1.0 cm^2^), and an Ag/AgCl/KCl saturated reference electrode. An SWV positive and negative sweep were performed using a CHI660C electrochemical work station, and voltammetric data were acquired. The schematic diagram for experimental procedure is found in the Supplementary Materials (see Figure S1).

## 3. Results and Discussion

### 3.1. Structural Characterization

**Figure 1:**
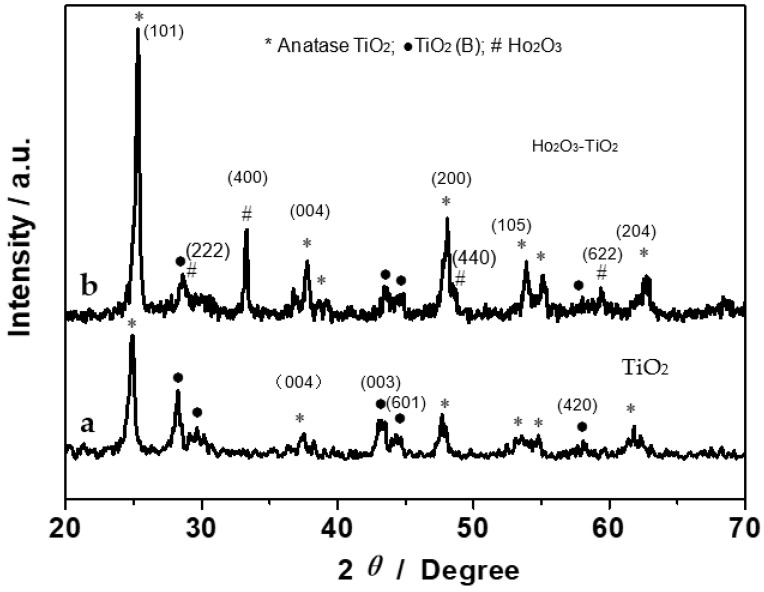
XRD patterns of (a) TiO_2_ and (b) Ho_2_O_3_-TiO_2_ nanobelts.

The synthesized nanobelts were first characterized by XRD measurements. Figure 1 shows the XRD patterns of (a) TiO_2_ and (b) Ho_2_O_3_-TiO_2_ nanobelts. It can be seen that TiO_2_-(B) (black dots) and anatase (asterisks) coexist in the TiO_2_ nanobelts (curve a) [20]; and for the Ho_2_O_3_-TiO_2_ nanobelts (curve b), the diffraction peaks appear at 2θ = 29, 33, 48 and 59 (denoted by #) that can be assigned to the Ho_2_O_3_ (222), (400), (440) and (622) facets (JCPDS 74-1984) [19]. Moreover, it can be seen that the diffraction peaks of Ho_2_O_3_-TiO_2_ are overall stronger than those of TiO_2_, indicating the enhanced crystallinity of the former. For example, as compared to curve a, curve b shows a strong and narrow TiO_2_ (004) diffraction peak, suggesting more {001} facets with a high surface energy in the Ho_2_O_3_-TiO_2_ sample [21,22].

**Figure 2:**
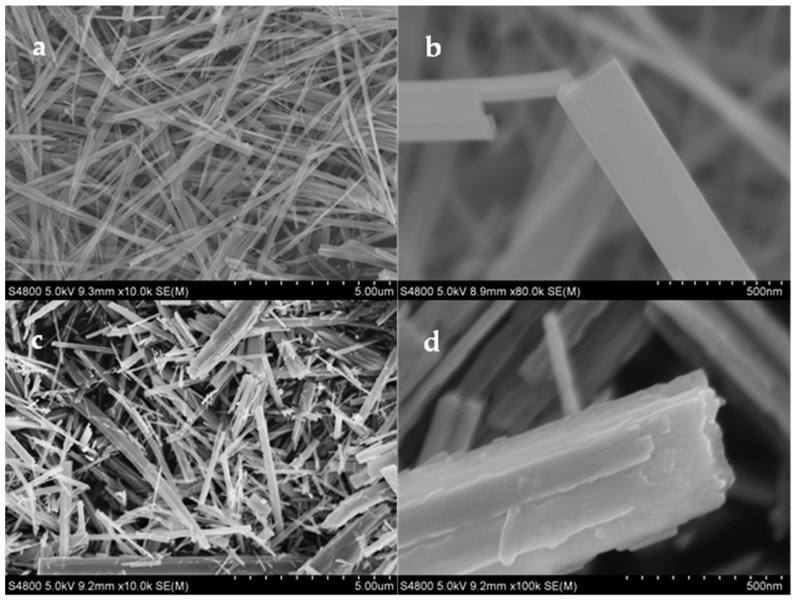
Representative SEM micrographs of (**a**,**b**) TiO_2_ and (**c**,**d**) Ho_2_O_3_-TiO_2_ nanobelts.

FESEM measurements were then carried out to investigate the nanobelts’ morphology and microstructure. The morphology of the resulting sample exhibited a drastic change, as Ti^4+^ was partly replaced by Ho^3+^. Compared to the TiO_2_ nanobelts (Figure 2a,b), the Ho_2_O_3_-TiO_2_ nanobelts were rougher, thinner, and shorter (Figure 2c,d). Usually, TiO_2_ nanobelts grow along the c-axis of the anatase lattice, which makes them lack the {001} facets (Figure 2a,b). As depicted in Figure 1, the TiO_2_ sample showed a relatively low and broad (004) peak. On the contrary, Ho_2_O_3_-TiO_2_ nanobelts displayed a multiple-layer structure and were much thinner than TiO_2_ nanobelts, and the surface was roughened, resulting in a significant increase in the effective concentration of the {001} facets (Figure 2c,d). This is consistent with the enhanced (004) diffraction peak in Figure 1b. As Ti^4+^ was partly replaced by Ho^3+^, oxygen vacancies might be produced in the Ho_2_O_3_-TiO_2_ nanocrystals, and facilitated the transport of oxygen-containing species as an active material.

### 3.2. Specific and Sensitive Detection of Cancer miRNAs

**Figure 3:**
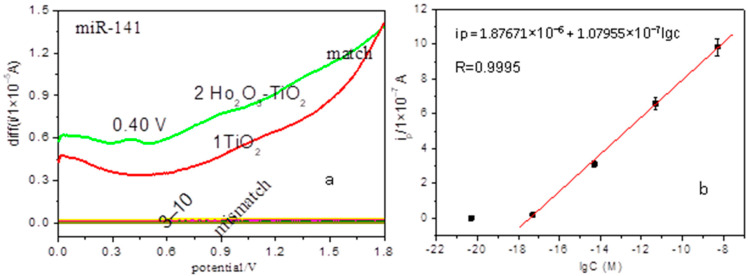
Electrochemical detection of cancer miRNAs. (**a**) SWV curves for miR-141: (1) match at the TiO_2_ nanobelts modified electrode; match (2) and mismatch (3–10) at the Ho_2_O_3_-TiO_2_ nanobelts modified electrodes; (**b**) Linear plot of anodic peak current (i_pa_) versus concentration of perfect match miR-141 ranging from 0 to 5 nM at Ho_2_O_3_-TiO_2_ nanobelts-modified electrodes.

**Figure 4:**
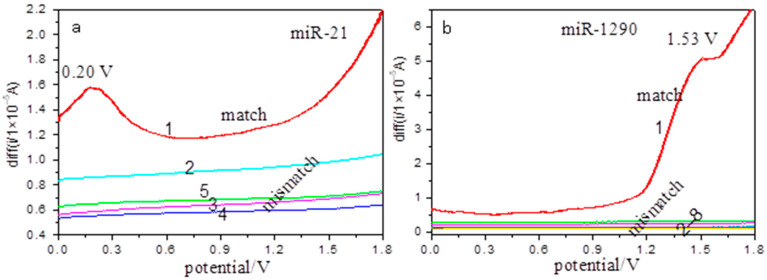
Electrochemical detection of cancer miRNAs at the Ho_2_O_3_-TiO_2_ nanobelts-modified electrodes. SWV curves for (**a**) miR-21: match (1) and mismatch (2–5); (**b**) miR-1290: match (1) and mismatch (2–8).

As is well-known, miR-141, miR-21, and miR-1290 are specific biomarkers of prostate cancer, osteosarcoma, and pancreatic cancer, respectively. The Ho_2_O_3_-TiO_2_ nanobelts prepared above were then used as a sensing electrode to detect these cancer microRNAs (Figure 3 and Figure 4) by using square-wave voltammetry (SWV). Compared to other electrochemical techniques, SWV possesses the highest sensitivity and specificity in electrochemical pulse techniques.

Figure 3a shows the SWV positive sweep curves of the perfect match and mismatch for 5 fM prostate cancer miR-141 (related sequences see Table 1) hybridization solutions at the TiO_2_ and Ho_2_O_3_-TiO_2_ nanobelts modified electrodes. As compared with the featureless response at the TiO_2_ nanobelts-modified electrode (Figure 3a(1)), there is a main anodic peak at +0.4 V and two minor ones at +0.9 V and +1.4 V, (Figure 3a(2)), corresponding to the electro-oxidation of perfect match miR-141 at the Ho_2_O_3_-TiO_2_ nanobelts-modified electrodes, which indicates the enhanced electrochemical sensing of the miRNA by the Ho_2_O_3_-TiO_2_ nanobelts. Additionally, for the Ho_2_O_3_-TiO_2_ nanobelts-modified electrodes, no oxidation peak appears for all mismatch solutions within the potential range (Figure 3a(3–10)), which indicates that Ho_2_O_3_-TiO_2_ nanobelts can selectively detect prostate cancer miRNA. The better sensing activity of Ho_2_O_3_-TiO_2_ nanobelts can be explained by results from the XRD (Figure 1b) and FESEM (Figure 2c,d) measurements, where the {001} facets of Ho_2_O_3_-TiO_2_ nanobelts were evidently increased due to the doping of Ho_2_O_3_. As is well known, anatase TiO_2_ {001} facets are active for oxygen adsorption with a high activity [23]. As mentioned earlier, Ho_2_O_3_-TiO_2_ nanobelts consisted of multiple layers and were much thinner than TiO_2_ nanobelts, and had a roughened surface. This resulted in a significant increase of the effective concentration of the {001} facets and facilitated oxygen adsorption on the Ho_2_O_3_-TiO_2_ nanobelt surface, leading to the enhanced electroxidation of miR-141 at the Ho_2_O_3_-TiO_2_ nanobelts electrodes [23]. Note that oxygen adsorption was not favored on stoichiometric anatase TiO_2_ {001}, but can be enhanced by the introduction of oxygen vacancy [24]. Compared with the TiO_2_ nanobelts, Ho_2_O_3_-TiO_2_ nanobelts actually contained abundant oxygen vacancies due to the partial replacement of Ti^4+^ by Ho^3+^ [25], consistent with the enhanced sensing performance.

Here, taking miR-141 as an example, we estimated the limit of detection of the Ho_2_O_3_-TiO_2_ nanobelts modified electrode. Different concentrations of perfect match miR-141 were detected at the Ho_2_O_3_-TiO_2_ nanobelts modified electrodes by using SWV measurements (see Figure S2). The variation of the anodic peak current (i_pa_) versus concentration of perfect match miR-141 was shown in Figure 3b, which exhibited a linear correlation, i_pa_(A) = 1.87671 × 10^−6^ + 1.07955 × 10^−7^ log(c) (r^2^ = 0.9995) for the perfect match miR-141 within the concentration range of 0 to 5 nM. The i_pa_ of the blank sample was 1.2944 × 10^−9^ A at 0.4 V, so the final limit of detection was estimated to be 4.2658 × 10^−18^ M (or 4.26 aM). The sensitivity is higher than results previously reported for miRNA nano-biosensors [26].

Under the same experimental conditions, detection of 0.9 μM osteosarcoma miR-21 and pancreatic cancer miR-1290 (related sequences in Table 2 and Table 3) were also tested with the Ho_2_O_3_-TiO_2_ nanobelts-modified electrodes (Figure 4). From Figure 4, it can be seen that the perfect match miR-21 and miR-1290 showed an oxidation peak at +0.2 V (Figure 4a) and +1.53 V (Figure 4b), respectively. Similarly, no oxidation peaks appear for all mismatch solutions within the same potential range (Figure 4a(2–5),b(2–8)). These results further confirmed that the Ho_2_O_3_-TiO_2_ nanobelts-modified electrodes could selectively detect cancer miRNAs at distinctly different potentials.

The high selectivity of miRNA detections by Ho_2_O_3_-TiO_2_ nanobelts can be explained within the context of the molecular structure. MiR-141, miR-21, and miR-1290 exhibit different base sequences and different molecular structures. They can form different hybrid bistrand structures with their respective match probe, which exhibits electrooxidation peaks at different potentials [7]. As shown in Figure 3 and Figure 4, the different cancer miRNA (i.e., miR-141, miR-21, and miR-1290) showed specific voltammetric features, suggesting that cancer miRNAs can be specifically detected via electrochemical potential sensing. Similarly, the perfect match and mismatch of these miRNAs have different hybrid bistrand structures. Compared with the perfect match, the mismatch nucleobases are exposed outside of the hybrid bistrand, resulting in different interactions between the miRNAs hybrid molecules and the electrode surface. Thus, the perfect match and mismatch of the cancer miRNAs exhibited a different electrochemical response, which was amplified by the formation of oxygen vacancy in Ho_2_O_3_-TiO_2_ [24]. 

### 3.3. Mechanism of the Electrochemical Detection of Cancer

**Scheme 1:**
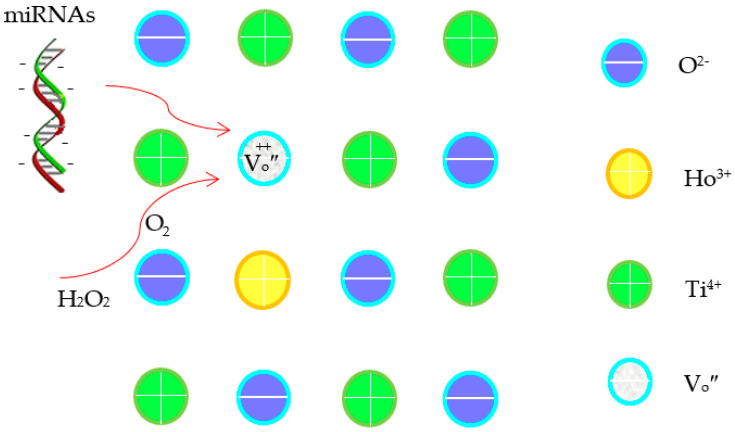
The illustration for a mechanism of the electrochemical detection of cancer miRNAs.

Within the present potential range, oxygen reduction reaction is inevitable (see Figure S3) [27]. Generally, the electrochemical reaction of O_2_ in aqueous media can occur via a pathway involving intermediates such as H_2_O_2_ and O_2_ (Equations (1)–(3)) [27,28]. The strong oxidants can enhance the electro-oxidation of miRNAs on the electrode surface. In comparison with TiO_2_ nanobelts, the multiple layers and roughened surface of the Ho_2_O_3_-TiO_2_ nanobelts effectively increased not only the specific surface area but also the concentration of the {001} facets (Figure 2), which further increased the active sites and promoted oxygen adsorption. Additionally, oxygen vacancies were formed in the Ho_2_O_3_-TiO_2_ nanobelts since Ti^4+^ was partially replaced by Ho^3+^ (Equation (4)), and promoted oxygen adsorption on the anatase TiO_2_ surface [24] as well as the diffusion of oxygen ions [29]. These collectively improved the surface electrocatalytic properties of the sensing electrodes [30]. Note that oxygen vacancies induced the surface adsorption of oxygen species in different states, i.e., the O-O bond in the superoxide state (O_2_^−•^) was oriented parallel to the surface along the {010} facet, while the peroxide state (O_2_^2−^) was perpendicular to the surface along the {001} facet, which changed the microenvironment of the sensing electrode surface. Thus, it affects the sensitivity and specificity of the sensing electrodes. Additionally, as the oxygen vacancies are positively charged while miRNAs are negatively charged, the oxygen vacancies can promote the miRNAs adsorption and the oxygen species transfer (Scheme 1) [31,32], leading to enhanced electrochemical detection performance.

Additionally, for the mismatched miRNA moleculars, the exposed mismatch bases increase the molecular steric hindrance, and nucleobases have hydrophobicity, all of which affect the mismatched miRNA moleculars’ adsorption on the Ho_2_O_3_-TiO_2_ electrode surface, resulting in their poor electrochemical sensing response.

(1) $$O_{2}+2H^{+}+2e^{-}\;\to {\;H}_{2}O_{2}$$

(2) $$H_{2}O_{2}+2H^{+}+2e^{-}\to 2H_{2}O$$

(3) $$H_{2}O_{2}\to 2H_{2}O+O_{2}$$

(4) $${\mathrm{Ho}}_{2}O_{3}\overset{2{\mathrm{TiO}}_{2}}{\to }2{\mathrm{Ho}}_{\mathrm{Ti}}^{\text{’}}+V_{o}^{\text{”}}+3O_{o}$$

## 4. Conclusions

In this work, Ho_2_O_3_-TiO_2_ nanobelts were successfully prepared and used as sensing active materials to detect cancer miRNAs. XRD and FESEM measurements indicated an efficient increase of the {001} facets in the nanobelts due to Ho_2_O_3_ doping. Electrochemical measurements showed that Ho_2_O_3_-TiO_2_ nanobelts could selectively and sensitively detect cancer miRNAs. These results suggest that Ho_2_O_3_-TiO_2_ nanobelts may serve as promising active materials for biosensing applications of early cancer diagnosis, drug screening, and molecular biology research.

## Figures and Tables

**Table 1:** Matching and mismatching sequences for prostate cancer miR-141.

No.	miRNA	Squences
1,2	miR-141 Match probe Mismatch probes	5′-UAACACUGUCUGGUAAGAUGG-3′ 5′-CCATCTTTACCAGACAGTGTTA-3′
3		5′-ACATCTTTACCAGACAGTGTTA-3′
4		5′-GCATCTTTACCAGACAGTGTTA-3′
5		5′-TCATCTTTACCAGACAGTGTTA-3′
6		5′-CCATCTTTACCCGACAGTGTTA-3′
7		5′-CCATCTTTACCGGACAGTGTTA-3′
8		5′-CCATCTTTACCTGACAGTGTTA-3′
9		5′-CCATCTTTACCAGACAGTGTTC-3′
10		5′-CCATCTTTACCAGACAGTGTTG-3′

Note: mismatched nucleobases are underlined. The serial number in Table 1 is consistent with Figure 3a.

**Table 2:** Matching and mismatching sequences for osteosarcoma miR-21.

No.	miRNA	Squences
1	miR-21 Match probe Mismatch probes	5′-TAGCTTATCAGACTGATGTTGA-3′ 5′-TCAACATCAGTCTGATAAGCTA-3′
2		5′-TCAACATCAGTGTGATAAGCTA-3′
3		5′-TCAACATCAGTTTGATAAGCTA-3′
4		5′-TCAACATCAGTCTGATCAGCTA-3′
5		5′-TCAACATCAGTCTGATAAGATA-3′

Note: mismatched nucleobases are underlined. The serial number in Table 2 is consistent with Figure 4a.

**Table 3:** Matching and mismatching sequences for pancreatic cancer miR-1290.

No.	miRNA	Squences
1	miR-1290 Match probe Mismatch probes	5′-TGGATTTTTGGATCAGGGA-3′ 5′-TCCCTGATCCAAAAATCCA-3′
2		5′-TCCCTGATCAAAAAATCCA-3′
3		5′-TCCCTGATCGAAAAATCCA-3′
4		5′-TCCCTGATCTAAAAATCCA-3′
5		5′-TCGCTGATCCAAAAATCCA-3′
6		5′-TCCCGGATCCAAAAATCCA-3′
7		5′-TCCCTGATCCAAACATCCA-3′
8		5′-TCCCTGATCCAAAAATACA-3′

Note: mismatched nucleobases are underlined. The serial number in Table 3 is consistent with Figure 4b.

## Data Availability

Not applicable.

## Supplementary Materials

The following supporting information can be downloaded at: https://www.mdpi.com/article/10.3390/bios12100800/s1.
