# Supplementary material for: Ho2O3-TiO2 Nanobelts Electrode for Highly Selective and Sensitive Detection of Cancer miRNAs
Source: Biosensors (Basel). 2022 Sep 27;12(10):800. doi: 10.3390/bios12100800 (PMC9599087; doi:10.3390/bios12100800)
Supplement: Supplementary file 1 [file biosensors-12-00800-s001.zip › biosensors-1825702-supplementary.pdf]

biosensors

MDPI

Supplementary Materials

Ho_2O_3-TiO_2 Nanobelts Electrode for Highly Selective and Sensitive Detection of Cancer miRNAs

Jingjie Cui [1,*], Xuping Wang [1] and Shaowei Chen [2]

1 School of Automation, Hangzhou Dianzi University, Hangzhou 310018, China
2 Department of Chemistry and Biochemistry, University of California, 1156 High Street, Santa Cruz, CA 95064, USA
* Correspondence: cuijingjie@hdu.edu.cn; Tel.: +86-57186878667

1. The Schematic Diagram for Experimental Procedure

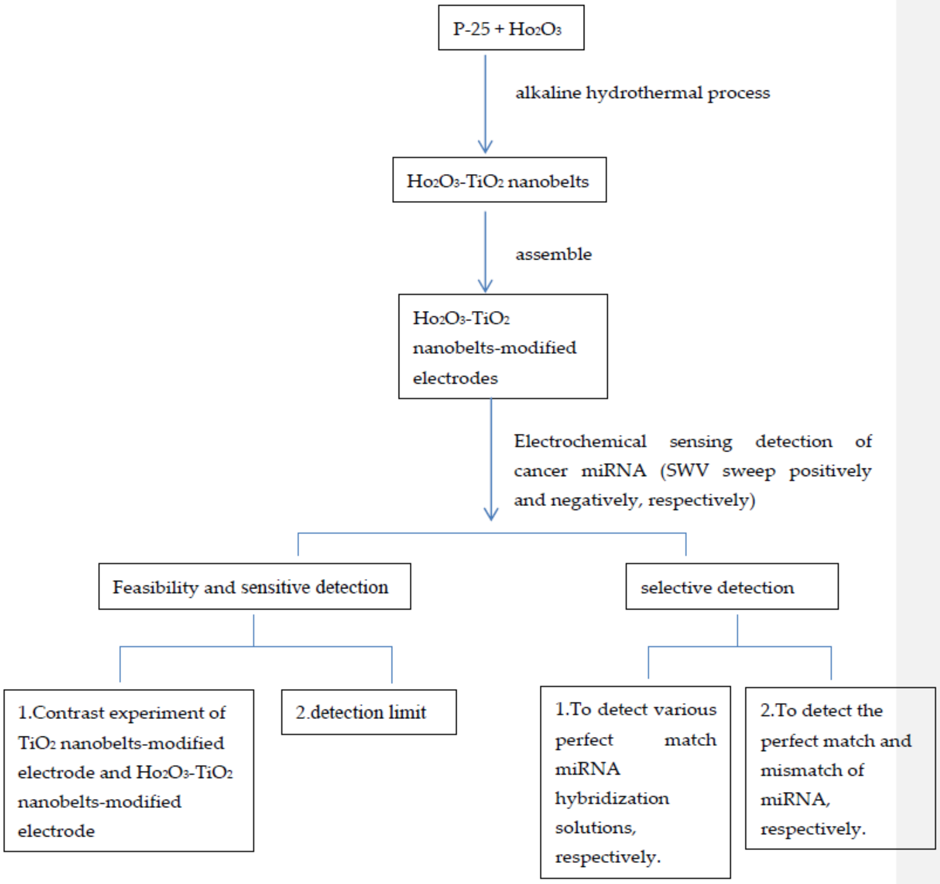

Figure S1. The schematic diagram for experimental procedure of detection of cancer miRNAs with a biosensing electrode based on Ho_2O_3-TiO_2 nanobelts.

2. Constructing the ipa-logc Figure

For the linear detection of miRNA, it seems complicated, our work shows that the linear results are not ideal. We guess that it may be related to the complex structure and electrochemical reaction process of miRNA in solution system, e.g. Figure S2.1 and Figure S2.2 are SWV curves for 5 fM and 5 aM miR-141 match at the Ho_2O_3-TiO_2 nanobelts modified electrode, respectively. All have an anodic peak at 0.4 V or so. However, if you plot them in the same figure, SWV curve for 5 aM miR-141 match only show a straight line (Figure S2.3). So it is inappropriate to put SWV for different concentrations of

miR-141 in the same figure. Our idea is to read ipa corresponding to each concentration sample (Table S2.1), owing to ip_a-c figure (Figure S2.4) is not able to help us to obtain the limit of detection, therefore, we construct the ipa-logc figure (Figure S2.5), that is, the Figure 3b in our manuscript, the ipa-logc figure show positively correlated between ip_a and logc in the detection range.

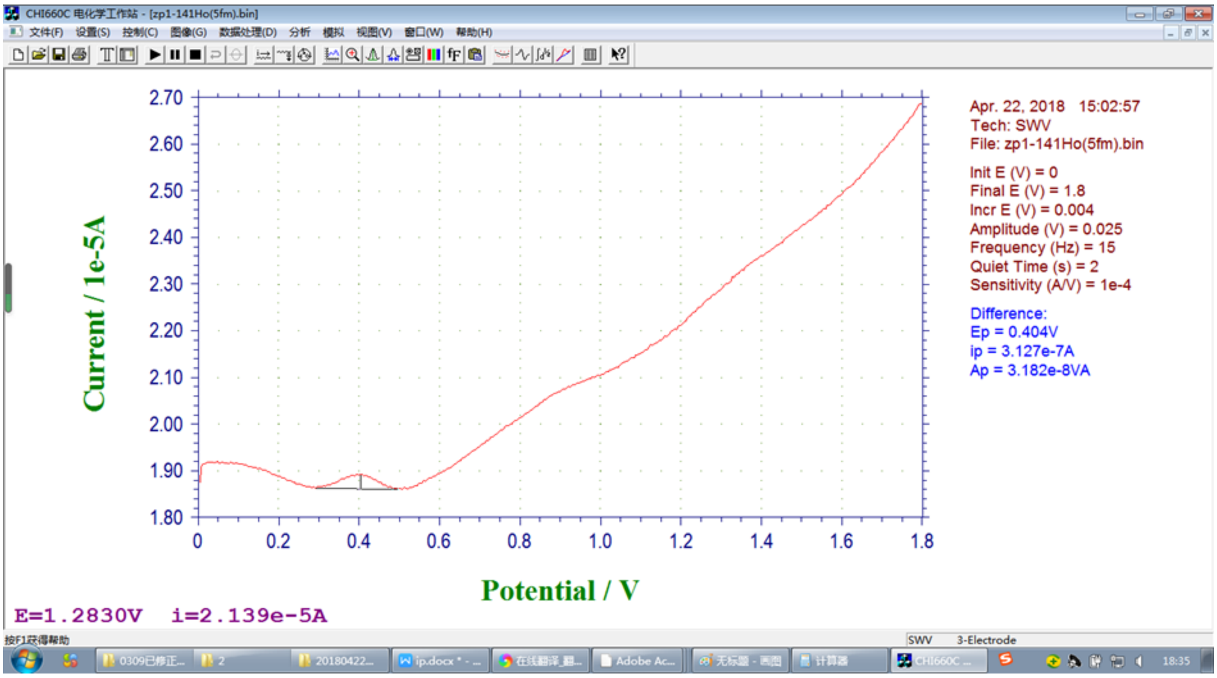

Figure S2.1 SWV curve for 5 fM miR-141 match at the Ho_2O_3-TiO_2 nanobelts.

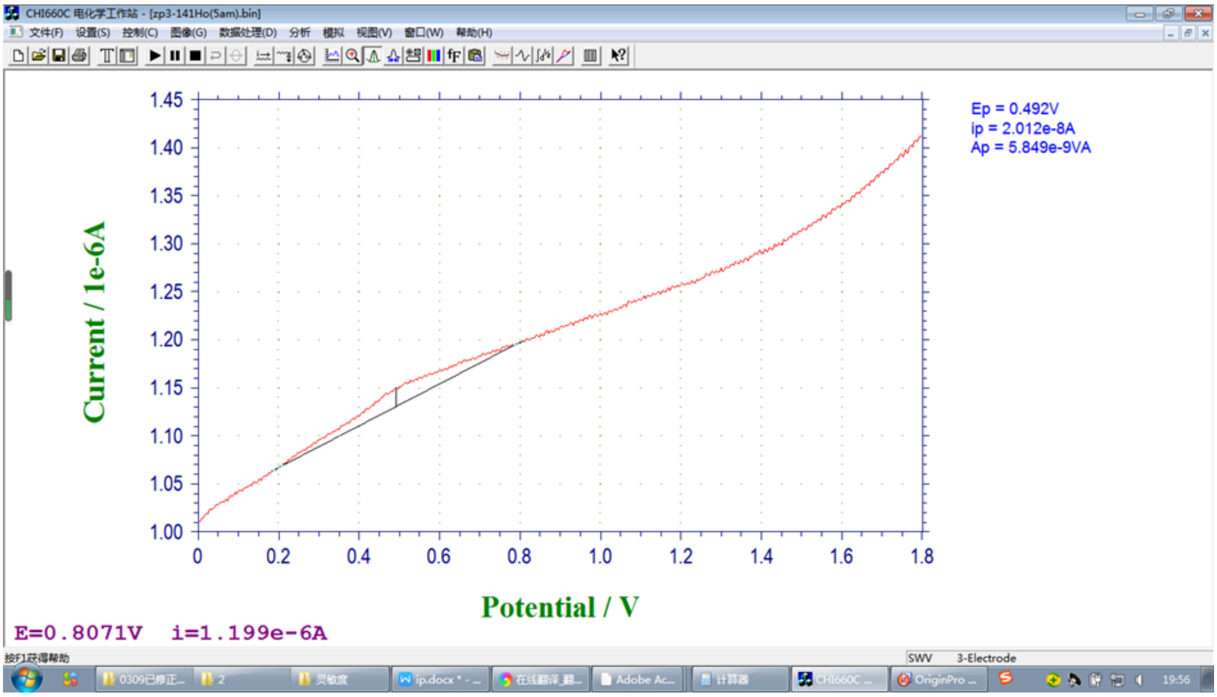

Figure S2.2 SWV curve for 5 aM miR-141 match at the Ho_2O_3-TiO_2 nanobelts modified electrode.

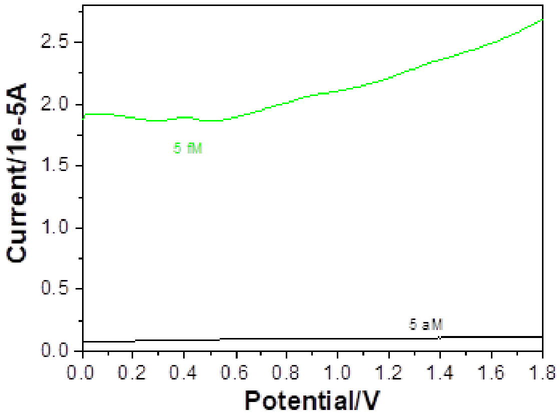

Figure S2.3 SWV curves for 5 fM and 5 aM miR-141 match at the Ho_2O_3-TiO_2 nanobelts modified electrodes.

Table S1 The ipa of different concentrations of perfect match miR-141 at the Ho_2O_3-TiO_2 nanobelts modified electrodes

C	5 nM	5pM	5fM	5 aM	0.005aM
i_{pa}/A	9.849e-7	6.592e-7	3.127e-7	2.012e-8	8.446e-10

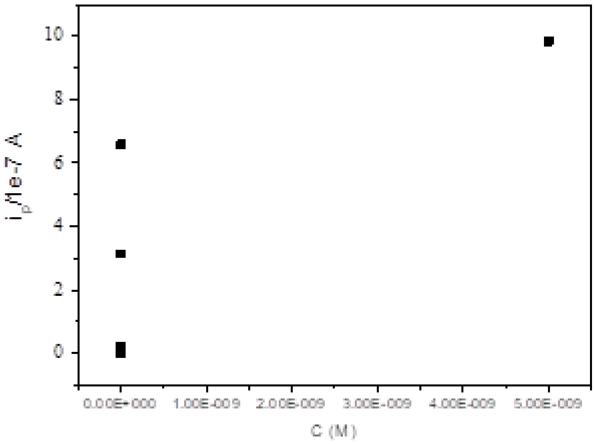

Figure S2.4 ipa-C.

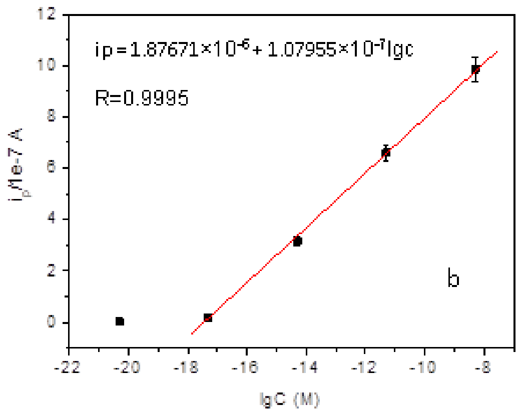

Figure S2.5 ipa-lgC, it is the Figure3b in our manuscript.

3. Oxygen Reduction Reaction

$$O_2 + 2H^+ + 2e^- \rightarrow H_2O_2 \qquad 0.2\ V \qquad (1)$$

$$H_2O_2 + 2H^+ + 2e^- \rightarrow 2H_2O \qquad 1.15\ V \qquad (2)$$

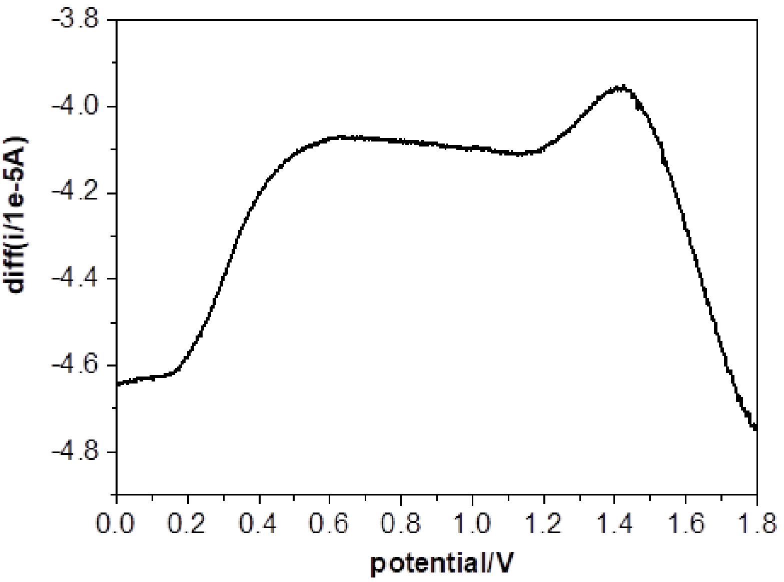

Figure S3. Electrochemical detection of cancer miRNAs at the Ho_2O_3-TiO_2 nanobelts modified electrodes. SWV sweep curves negatively for the perfect match miR-21 solution. The results of miR-1290 and miR-141 are similar to that of miR-21 (data not shown).
